# Arrhythmia Evaluation in Wearable ECG Devices

**DOI:** 10.3390/s17112445

**Published:** 2017-10-25

**Authors:** Muammar Sadrawi, Chien-Hung Lin, Yin-Tsong Lin, Yita Hsieh, Chia-Chun Kuo, Jen Chien Chien, Koichi Haraikawa, Maysam F. Abbod, Jiann-Shing Shieh

**Affiliations:** 1 Department of Mechanical Engineering and Innovation Center for Big Data and Digital Convergence, Yuan Ze University, Taoyuan, Chung-Li 32003, Taiwan; muammarsadrawi@yahoo.com; 2 Healthcare and Beauty RD Center, Kinpo Electronics, Inc., New Taipei City 222, Taiwan; lance_lin@kinpo.com.tw (C.-H.L.); lotusytlin@calcomp.com.tw (Y.-T.L.); yita_hsieh@calcomp.com.tw (Y.H.); cckuo@calcomp.com.tw (C.-C.K.); jcchien@kinpo.com.tw (J.C.C.); koichi_h@kinpo.com.tw (K.H.); 3 Department of Electronic and Computer Engineering, Brunel University London, Uxbridge UB8 3PH, UK; Maysam.Abbod@brunel.ac.uk

**Keywords:** wearable sensor, arrhythmia, sample entropy, fast Fourier transform, artificial neural networks

## Abstract

This study evaluates four databases from PhysioNet: The American Heart Association database (AHADB), Creighton University Ventricular Tachyarrhythmia database (CUDB), MIT-BIH Arrhythmia database (MITDB), and MIT-BIH Noise Stress Test database (NSTDB). The ANSI/AAMI EC57:2012 is used for the evaluation of the algorithms for the supraventricular ectopic beat (SVEB), ventricular ectopic beat (VEB), atrial fibrillation (AF), and ventricular fibrillation (VF) via the evaluation of the sensitivity, positive predictivity and false positive rate. Sample entropy, fast Fourier transform (FFT), and multilayer perceptron neural network with backpropagation training algorithm are selected for the integrated detection algorithms. For this study, the result for SVEB has some improvements compared to a previous study that also utilized ANSI/AAMI EC57. In further, VEB sensitivity and positive predictivity gross evaluations have greater than 80%, except for the positive predictivity of the NSTDB database. For AF gross evaluation of MITDB database, the results show very good classification, excluding the episode sensitivity. In advanced, for VF gross evaluation, the episode sensitivity and positive predictivity for the AHADB, MITDB, and CUDB, have greater than 80%, except for MITDB episode positive predictivity, which is 75%. The achieved results show that the proposed integrated SVEB, VEB, AF, and VF detection algorithm has an accurate classification according to ANSI/AAMI EC57:2012. In conclusion, the proposed integrated detection algorithm can achieve good accuracy in comparison with other previous studies. Furthermore, more advanced algorithms and hardware devices should be performed in future for arrhythmia detection and evaluation.

## 1. Introduction

Nowadays, wearable sensor-based system has been applied to wide applications. Fall and activity monitoring study with utilizing wearable sensor has been conducted by Shany et al. [1]. This kind of system was also used for Parkinson’s disease with the combination of Support Vector Machine (SVM) by a study conducted by Patel et al. [2]. Meanwhile, Corbishley et al. used non-invasive and continuous wearable system for breathing monitoring [3]. Furthermore, wearable electrocardiogram (ECG) device was also utilized for the emotion classifications via hear rate variability [4].

Recently, the implementation of intensively evaluated ECG signal through wearable sensor is one of the essential issues for the cardiovascular-related diseases. For example, arrhythmia has been one of the concerning cardiac diseases. Some of the arrhythmia cases are classified as life-threatening events. Therefore, Rosenberg et al. utilized long-term monitoring system for atrial fibrillation monitoring [5]. A study by Baig et al. evaluated wearable ECG system for older-adult population [6]. Furthermore, Fensli et al. utilized wearable sensor for arrhythmia detection applied to tele-home care system via general packet radio service (GPRS) to personal computer as base station. This information will be evaluated by doctors with remote system for the rhythm evaluation by the internet connection server [7]. Lin et al. developed an intelligent telecardiology system for sinus tachycardia, sinus bradycardia, wide QRS complex, atrial fibrillation, and cardiac asystole [8]. This system can also activate emergency alarm. In advanced, Hu et al. had successfully applied hidden Markov model to using wearable system for ECG arrhythmia evaluation [9]. Meanwhile, recent study by Hadiyoso et al. also conducted a study on arrhythmia detection via smart phone [10].

Atrial fibrillation (AF) and ventricular fibrillation (VF) are frequent arrhythmias. The former, AF, is one of the arrhythmias related to age and has serious effect on morbidity, mortality, and cost [11]. AF also is an independent factor and has significant effect on the risk of stroke by a study conducted by Wolf et al. on five thousand cases both female and male for more than thirty years [12]. Kara et al. utilized power spectral density and Daubechies wavelets with backpropagation artificial neural network (ANN) for AF detection [13]. Roonizi et al. used extended Kalman filter to evaluate AF frequency [14]. A study by Mohebbi et al. investigated AF by applying the feature dimension reduction technique with SVM classifier [15]. Abdul-Kadir et al. used dynamic ECG system according to second order differential equation of ECG behavior with cross validation technique by utilizing SVM and ANN as predictors [16]. Recently, Rajpurkar et al. have utilized one of the deep learning techniques, namely a 34-layer convolutional neural network for detecting arrhythmia, including AF [17].

Another arrhythmia is VF. According to McWilliam, VF has strong correlation with sudden cardiac diseases [18] and it is critical to defibrillation [19]. Alonso-Atienza et al. have applied bootstrap resampling-based feature extraction SVM classifier for VF detection [20]. A study by Anas et al. describes how empirical mode decomposition method is used to discriminate VF and non-VF rhythms [21].

Atrial premature complex (APC) and ventricular premature complex (VPC) are other frequent arrhythmias that, according to the ANSI/AAMI EC57:2012, can be classified as supraventricular ectopic beat (SVEB) and ventricular ectopic beat (VEB). Research on detecting these conditions and other arrhythmias have been in several previous studies. Thong et al., have utilized paroxysmal atrial fibrillation for APC calculation [22]. Sayadi et al. have used extended Kalman filter for VPC detection [23]. Similarly, a study by Özbay et al. that evaluated the performance of the neural networks also performed the initialization of fuzzy C-means for APC, VPC, and other ECG arrhythmia [24]. Song et al. utilized support vector machine with the combination of dimensionality reduction using principal component analysis, and linear discriminant analysis for arrhythmia classifier including APC and VPC. They found better result as compared to multilayer perceptron (MLP) and fuzzy inference system [25].

However, these previous studies are relatively computationally complex to be applied to wearable devices. Several studies, which are relatively less computational complexity, performed well for AF and VF detection. For AF detection, Zhou et al. proposed a powerful algorithm for real time detection of AF. This study evaluated the heart rate to create a symbolic and word sequences. In advanced, Shannon entropy was utilized to evaluate the word sequence in order to classify AF [26]. On the other hand, FFT algorithm has been a robust algorithm for signal detection algorithm in recent studies [27,28,29,30] and has been effectively applied for VF detection for decades. For VF, Clayton et al. evaluated signal spectrum by utilizing fast Fourier transform (FFT) and maximum entropy for VF detection [31]. Afonso et al. applied short time Fourier transform (STFT), smoothed pseudo Wigner-Ville distribution and cone-shaped kernel for the VF evaluation [32]. Recently, wearable sensor-based system has been widely applied and utilized, the chance of the real-time evaluation for the arrhythmia detection with the less-complicated algorithms is highly likely an acceptable investigation. Hence, the main purpose of this study is to efficiently apply less complexity algorithms for real-time detection of arrhythmias utilizing wearable devices based on ANSI/AAMI EC57:2012 evaluation.

## 2. Materials and Methods 

**Figure 1:**
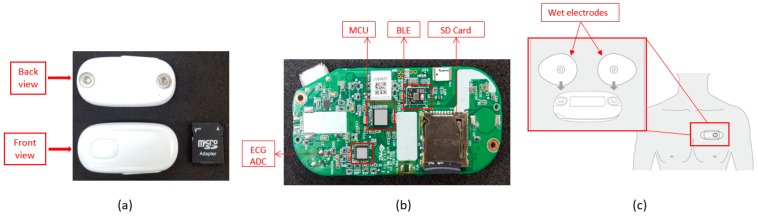
The BC1 electrocardiogram (ECG) device: (**a**) Front and back views; (**b**) Processing, transmitting and saving units detail; and, (**c**) Position with the electrodes.

For the hardware part, BC1 ECG device (Bio Clothing One, XYZ life BC1, Kinpo Inc., Taipei, Taiwan) single lead heart rate monitor front end is ADI ADS 8232 (Analog Devices, Inc., Norwood, MA, USA). The BC1 ECG device uses wet electrode. The detail of the BC1 ECG device is shown in Figure 1. Meanwhile, its specification is shown in Table 1. This device is configured by 0.5 Hz two-pole high-pass filter and two-pole 40 Hz for the low-pass filter [33]. For the micro controller unit (MCU), Texas Instruments MSP430 series is selected to have an ultra-low power unit that has 128 KB flash ROM and 8 kB SRAM. This unit is a 16-bit reduced instruction set computer (RISC) architecture of up to 25 MHz system clock with 12-bit analog-to-digital converter (ADC). In further, the Bluetooth low energy (BLE) using Texas Instruments CC25 series (Texas Instruments Incorporated, Dallas, 75243 TX, USA) connection system is utilized to have a power-optimized system-on-chip (SOC) solution that supports maximum 2 Mbps data rates. The small start button powers the device on. The device will detect the connection of the Bluetooth, which will either associate the smartphone or not. When there is no Bluetooth device connection, the device will be turned to off-line state allowing the data to be stored only in the SD card. Meanwhile, the on-line evaluation will send real-time ECG data to the smartphone application for the arrhythmia classification.

This study uses PhysioNet database [34] for algorithm development and testing. Furthermore, simulator data from Fluke ProSim 8 vital sign patient monitor simulator (Fluke Biomedical Division of Fluke Electronics Corporation, Everett, 98203 WA, USA) is conducted for real-time detection. The four databases provided by PhysioNet are American Heart Association database (AHADB), Creighton University Ventricular Tachyarrhythmia database (CUDB) [34,35], MIT-BIH Arrhythmia database (MITDB) [34,36], and MIT-BIH Noise Stress Test database (NSTDB) [34,37]. For SVEB (i.e., APC) classification, 44 records for MITDB is analyzed. Meanwhile for VEB (i.e., VPC) detection, 78 records for AHADB, 44 records for MITDB, and 12 records for NSTDB are used in the evaluation. For AF detection, 44 records for MITDB are utilized for the evaluation. Furthermore, 78 records for AHADB, 44 records for MITDB, and 35 records for CUDB are used for VF classification. Evaluation of sensitivity (Se), positive predictivity (+P), and false positive rate (FPR) are defined for the evaluation performance of SVEB (i.e., APC) and VEB (i.e., VPC). Meanwhile, episode sensitivity (ESe), episode positive predictivity (E + P), duration sensitivity (DSe), and duration positive predictivity (D + P) are utilized for AF and VF detections. All of the evaluations are performed according to ANSI/AAMI EC57:2012 [38].

For simulation and visualization in smartphone for real-time application, Fluke ProSim 8 is also utilized. Simulation data includes several ECG rhythms: normal sinus rhythm, APC, VPC, AF, and VF signals in real-time situation and evaluation on the smartphone. Initially, simulation data from Fluke simulator is transferred to BC1. Programming of the arrhythmia algorithm is conducted in Java (Android) and Objective-C (iOS). The performance of this algorithm is evaluated by WFDB (WaveForm DataBase) software on Windows acquired from PhysioNet.

**Figure 2:**
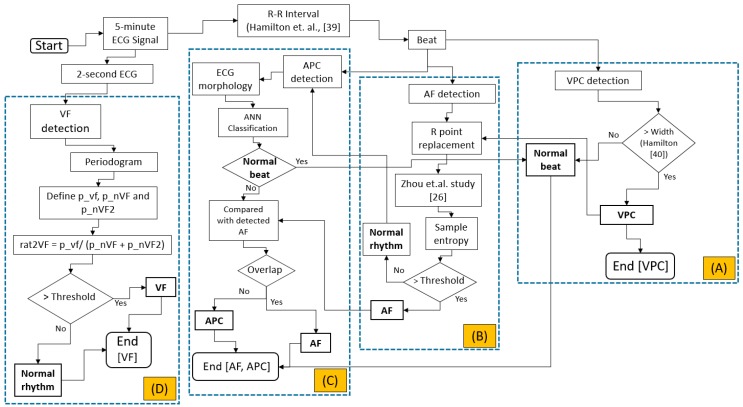
Integrated arrhythmia evaluation flowchart; (**A**) Ventricular premature complex detection; (**B**) Atrial fibrillation detection; (**C**) Atrial premature complex detection; (**D**) Ventricular fibrillation detection.

The integrated evaluation is started by evaluating the APC, VPC, AF, and VF based on ANSI/AAMI EC57:2012, as shown in Figure 2. Originally, the data downloaded from PhysioNet [34,35,36,37] is downsampled to 200 Hz. Initially the 5-min ECG signal is evaluated either based on the beat, for VPC, AF, and APC evaluations or the raw ECG signal, the 2-s window, for VF detection.

VPC evaluation is initiated by the R-R interval evaluation. The evaluation is calculated based-on the study by Hamilton et al. [39]. Another study by Hamilton [40] is utilized for VPC evaluation, as shown on Figure 2A. For AF calculation, the previous evaluation of VPC beat is essentially important. The abnormal beats (i.e., VPC beats) will imitate the R-R interval in the normal rhythm. This phenomenon highly likely increases the uncertainty in classifying either normal or AF rhythm. Previous classification results for detecting VPC will be utilized to reform the original R-R interval by averaging the previous beat and the next beat. The AF evaluation is originally calculated based on Zhou et al. study [26]. The heart rate is calculated from the original R-R interval. This heart rate is converted to symbolic sequence using Equation (1). Furthermore, this symbolic sequence is utilized for the word value by Equation (2) as shown by the following:

(1) $$sy_{n}=\;\{\begin{array}{cc} 63 & if\;hr_{n}\geq 315\\ \lfloor hr_{n}/5\rfloor & other\;cases \end{array}$$

(2) $$wv_{n}=\left(sy_{n-2}\;x\;2^{12}\right)+\left(sy_{n-1}\;x\;2^{6}\right)+\;sy_{n}$$

where hr is the heart rate, $sy_{n}$ is the symbolic sequence, and $wv_{n}$ is the word value. This word value sequence evaluation, originally calculated using Shannon entropy, is replaced by sample entropy algorithm [41]. AF evaluation can be seen in Figure 2B.

**Figure 3:**
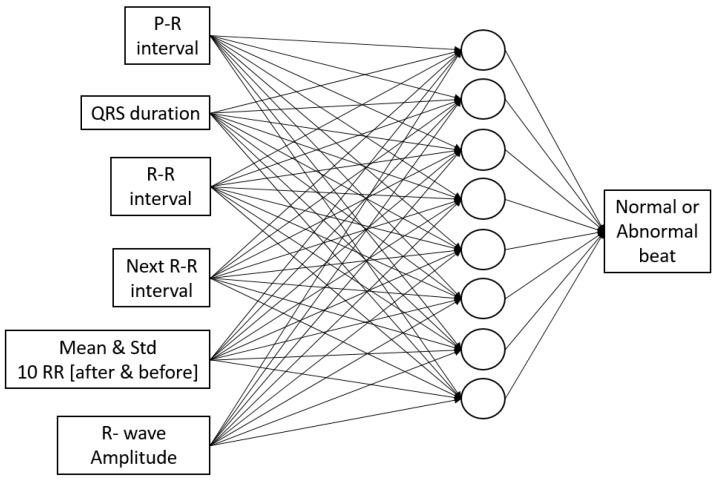
Artificial neural network (ANN) structure for detecting normal or abnormal beat in APC detection algorithm.

For APC detection, the morphological ECG is utilized for feature extraction and artificial neural networks. Multi-layer perceptron with backpropagation training algorithm and single hidden layer is utilized. Features for the ANN input extracted from ECG signal are P-R interval, QRS duration, R-R interval, next R-R interval, average, and standard deviation of R-R interval of 10 beats before and after the current beat, and R-wave amplitude, as shown in Figure 2C and Figure 3, which is based on our previous study [42]. The data is divided into 60% for training, 20% for validation, and 20% for testing.

After R-R interval-based algorithm is performed, the raw ECG signal-based evaluation is calculated. The initial 5-min ECG segment is reshaped to several 2-s ECG signals. This evaluation is organized to avoid mixed rhythms for the classification. The periodogram evaluation is utilized by finding its maximum point corresponding to the frequency of the shortened ECG segments. According to Lo et al., the dominant VF waveform frequency is between 1 Hz and 7 Hz [43]. Our study utilizes a similar range with some offset. Three focused area of maximas are defined. The first one is the VF area (p_vf). This area is located in between the frequency of greater than equals to 2.61 Hz and less than equals to 4.95 Hz. The next area is the first area of non-VF (p_nVF), which is between frequency greater than 0.5 Hz and less than 2.61 Hz. The last area is the second non-VF (p_nVF2), which is located between frequency greater than 4.95 Hz and less than or equals to 10 Hz. The ratio of the p_vf to the summation of the p_nVF and p_nVF2 is defined in order to classify either normal or VF rhythm. The threshold of the ratio is fixed to 3.96. The detailed evaluation of the VF arrhythmia can be seen in Figure 2D.

## 3. Results

**Figure 4:**
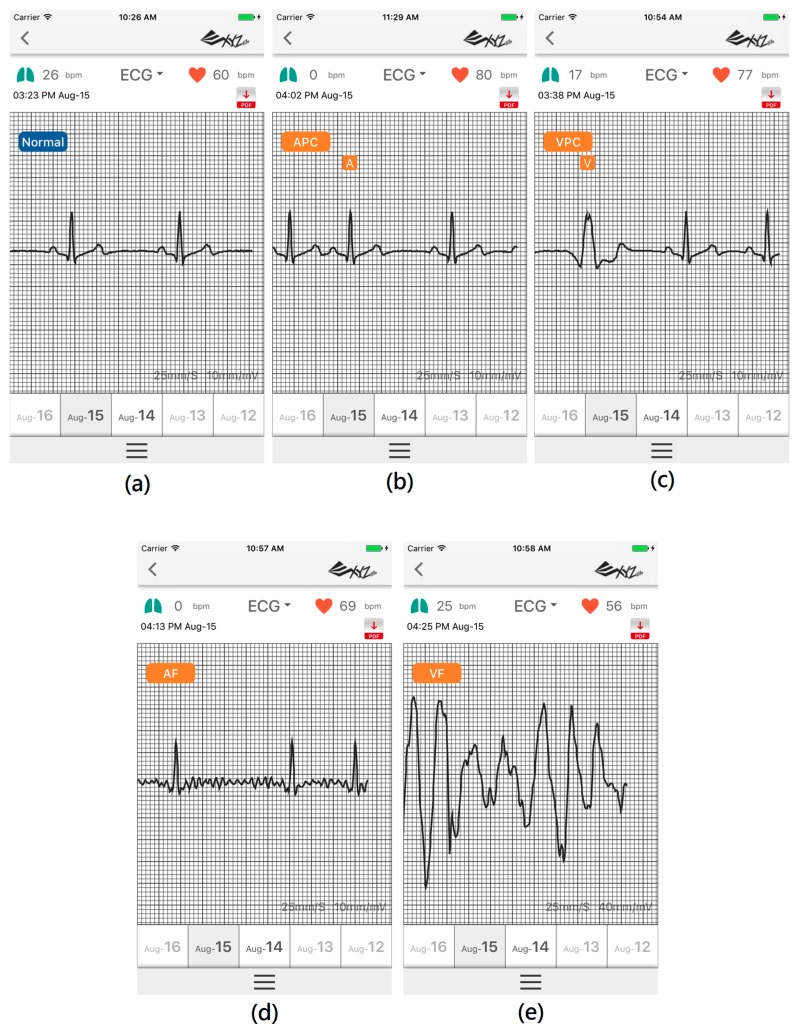
Simulation result from Fluke simulator displayed on mobile phone; (**a**) Normal sinus rhythm; (**b**) Atrial Premature Complex (APC); (**c**) Ventricular Premature Complex (VPC); (**d**) Atrial fibrillation; and, (**e**) Ventricular fibrillation.

**Figure 5:**
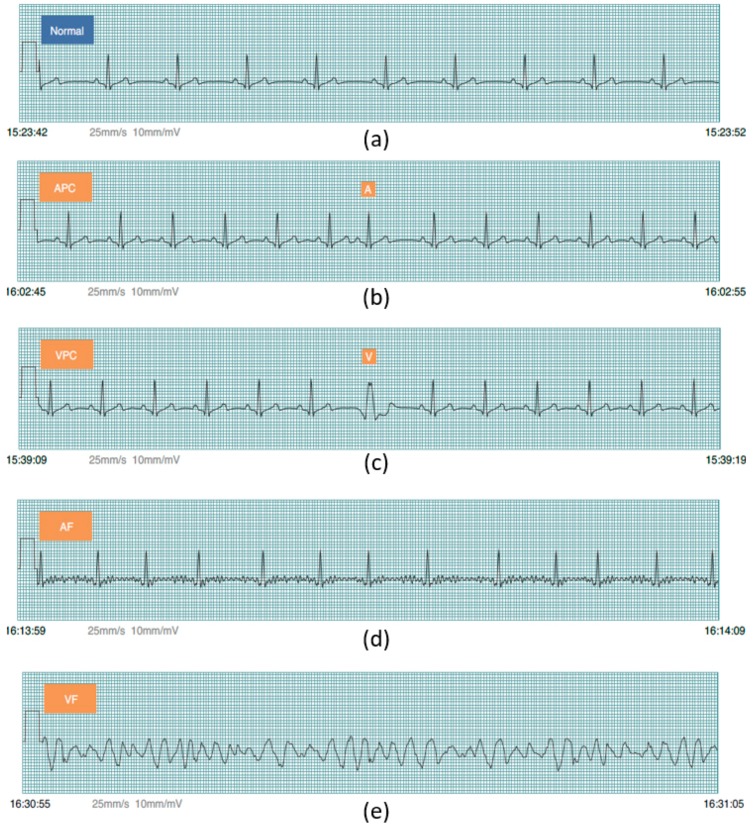
Documented simulation results from Fluke simulator; (**a**) Normal sinus rhythm; (**b**) Atrial Premature Complex (APC); (**c**) Ventricular Premature Complex (VPC); (**d**) Atrial fibrillation and (**e**) Ventricular fibrillation.

Prior to PhysioNet database evaluation, Fluke simulator data of Normal sinus rhythm, APC, VPC, AF, and VF are utilized from BC1 for algorithm evaluation on smartphone in real-time condition. Besides, visualizing real-time signals with its signal annotation are shown in Figure 4. For APC and VPC, detection is evaluated based-on the R-wave of the ECG signal. Meanwhile, normal sinus rhythm, AF and VF evaluation works based-on a segment. The mobile application is also able to store the documented signal with its signal annotation as the off-line evaluation records that can be seen in Figure 5.

The entire evaluation of arrhythmia can be seen in Table 2 and Table 3. For SVEB (i.e., APC) evaluation utilizing the MITDB database, the performances are 79.87%, 67.14%, and 1.323%, respectively, for the gross evaluation of Se, +P and FPR. Meanwhile for the average evaluation, Se, +P and FPR are 71.35%, 36.9%, and 2.098%, respectively.

The next evaluation is VEB (i.e., VPC). For this evaluation, utilizing a study by Hamilton [38], the gross evaluation from AHADB for Se, +P and FPR are 89.75%, 96.08%, and 0.371%, respectively. For average evaluation, Se, +P and FPR are 86.52%, 84.67%, and 0.458%. For MITDB database, the algorithm performances are 93.10%, 95.65% and 0.321% for gross evaluation of Se, +P, and FPR, respectively. Average evaluation has Se, +P, and FPR by 87.27%, 73.26%, and 0.336%, respectively. The third database for the VEB evaluation utilizes the NSTDB database. The performance for this database for gross evaluation respectively for the Se, +P and FPR are 83.22%, 45.79% and 10.180%. The average evaluation has 58.17%, 50.86% and 9.032% respectively Se, +P and FPR.

The next evaluation is for AF and VF rhythms. AF performance evaluation is calculated using MITDB database. For the gross evaluation, the performances are 62%, 100%, 92%, and 92% for ESe, E + P, DSe, and D + P, respectively. The average evaluations for ESe, E + P, DSe, and D + P, are 70%, 100%, 85%, and 86%.

The next evaluation is VF detection. This evaluation starts using AHADB database. For the gross evaluation, performances are 90%, 95%, 28%, and 97% for ESe, E + P, DSe, and D + P, respectively. Average evaluations for ESe, E + P, DSe, and D + P are 94%, 69%, 33%, and 70%. The second database used for VF evaluation is MITDB database. The gross evaluations are 100%, 75%, 69%, and 88% for ESe, E + P, DSe, and D + P, respectively. For average evaluation, it is 100%, 33%, 69%, and 33% for ESe, E + P, DSe, and D + P, respectively. The last database utilized for VF evaluation is CUDB. For this database, the gross evaluations are 83%, 90%, 32%, and 94% for ESe, E + P, DSe, and D + P, respectively. Meanwhile, for average evaluation it is 84%, 83%, 40%, and 84%, for ESe, E + P, DSe, and D + P, respectively.

## 4. Discussion

This study evaluates several arrhythmias, SVEB (i.e., APC), VEB (i.e., VPC), AF, and VF, based-on ANSI/AAMI EC57:2012 of totally 169 records from three PhysioNet databases with applying less computationally complicated algorithms. The performances of the algorithms are evaluated based-on the sensitivity, positive predictivity, and false positive rate. The applied methods utilized in this study are relatively less complex; namely sample entropy, FFT, and the ANN. For ANN, the features extracted from ECG signal are also acceptable in the feedforward run. This condition has purposed to minimize the computational time, while performing testing in the real-time application.

In order to study the measurement evaluation of previous studies conducted based-on ANSI/AAMI EC57, the results are compared to our results as shown on Table 4 for SVEB and VEB results. For SVEB and VEB, a study conducted by De Chazal et al. [44] is investigated. This study showed that SVEB evaluation produced gross evaluation of Se, +P and FPR as 75.9%, 38.5%, and 4.7%, respectively. For comparison purposes, we found that our study has better performances with respect to gross evaluation of Se, +P and FPR, which are 79.87%, 67.14%, and 1.323%, respectively. For VEB classification, De Chazal et al. [44] has gross evaluation of Se, +P and FPR are 77.7%, 81.9%, and 1.2%. Meanwhile, with utilizing a study by Hamilton [40], our results also showed better achievement by producing 93.1%, 95.65%, and 0.321%, respectively, for gross evaluation of Se, +P and FPR.

For AF study, results are compared with a previous study conducted by Young et al. [45]. This study performed hidden Markov model (HMM) evaluation using ANSI/AAMI:EC57 for evaluation. Twelve MIT-BIH Arrhythmia database records were utilized for training. Furthermore, for testing data, MIT-BIH AF database was used. In order to perform a comparison to this study, only training results from Young et al. study are investigated. From the results of Young et al. study, a sensitivity evaluation provides a better result of 97.7% as compared to this study evaluating 44 MIT-BIH Arrhythmia database records for ESe that has 62%, and DSe that has 92% for gross statistics evaluations. However, this study produces better results for both E + P that has 100%, and D + P that has 92% for gross statistics evaluations in comparison to their study, which resulted in 86.77% for the positive predictivity.

For a VF comparison study, studies by Park et al. [46] and Moraes et al. [47] are utilized. A study by Park et al. evaluated AHADB and MIT-BIH arrhythmia databases by applying decision rule-based algorithm and utilizing ANSI/AAMI:EC57. This study has evaluated the duration sensitivity and duration positive predictivity. For AHADB evaluations, a study by Park et al., utilized 11 records from AHADB, have 98.1% and 99.1%, respectively, for the DSe and D + P results. Meanwhile, our study, evaluating 78 records from AHADB, for DSe and D + P produces 28% and 97%, respectively for gross statistics evaluations. Furthermore, for MIT-BIH arrhythmia database evaluation, a study by Park et al., evaluated only record 207, has 88.5% and 86.3% for DSe and D + P, respectively. Hence, this study, utilizing 44 records, has achieved 69% and 88% for DSe and D + P, respectively.

Another study is performed for the purpose of VF evaluation comparison. Moraes et al. [47] conducted a study by combining two algorithms, VF filter leakage and complexity measure algorithms. This study has also utilized ANSI/AAMI:EC57 for the evaluation of CUDB. The combined algorithm by Moraes et al. study provided sensitivity and positive predictivity evaluations, by utilizing 30 records. For comparison study purposes, our results, utilizing 35 records, have 83% for ESe and 32% for DSe gross statistics evaluations. Meanwhile, a study by Moraes et al. has 70.32% for the sensitivity. In addition, the results of this study have 90% for E + P and 94% for D + P gross statistics evaluations. However, study by Moraes et al. has 94.66% positive predictivity. The overall comparison of the PhysioNet-based database for the AF and VF can be seen by Table 5.

The evaluation of AF evaluation in the wearable ECG device is compared to study by Lin et al. [8]. This study applied the expert system algorithm. A three-lead ECG device with 512 Hz sampling frequency and 12-bit resolution was utilized for 10 normal and 20 AF patients. For the evaluation, the 12-lead ECG system result was investigated by the cardiologists. The sensitivity and the positive predictive performance are 94.56% and 99.39%, respectively. For the comparison, Lin et al. study performed better accuracy than our study. However, our study evaluates the episode and duration separately according to ANSI/AAMI:EC57 for the sensitivity and positive predictivity.

For computational time, a study by Chakroborty et al. is utilized for the comparison [48]. This study proposed a solution for the arrhythmia classifications. The classified arrhythmias are normal, left bundle branch block (LBBB), right bundle branch block (RBBB), PVC, and PAC. The MIT-BIH Arrhythmia database was utilized for the evaluation. This study provided the overall evaluation time is 6875.3 s. For this study in computational time evaluation, the personal computer (PC), and smartphone-based computational time are evaluated. The PC specification is MacBook Air, Intel Core i5, and 1.6 GHz. Meanwhile, the smartphone is iPhone 5S, A7 chip, 64-bit architecture, and 1.3 GHz frequency. The results show that the computational time for the PC-based calculation is about 174.802 s. Luckily, smartphone-based calculation produces 1840.791 s. The evaluation time in PC and smartphone for MIT-BIH Arrhythmia database can be seen in Table 6. On average, the PC-based computational is 3.591 s and the smartphone-based takes 41.836 s. Meanwhile, our proposed study has been shown less computational time, as shown in Table 7. However, this comparison may not be fully acceptable due to the comparison of LBBB and RBBB detections versus with AF and VF evaluations.

There are several limitations of this study. The first comes from the sliding window for detecting AF. In this study evaluation using sample entropy needs a huge number of the R-R interval. This condition makes delay for the evaluation. However, according to Logan et al., the 5-min sliding window is an acceptable wait for AF detection cases [49].

The second one, ideally, the evaluation of AF and VF should be performed at the same time. However, AF evaluation is performed based-on RR interval and VF evaluation, which is based-on raw ECG signal, these conditions will affect one another. For AF, when evaluation follows VF detection using the raw ECG segment, it will be in an RR shortage condition for the evaluation. Meanwhile, for VF, when the evaluation follows the AF detection (i.e., the R-R interval-based calculation), it highly likely mixes some rhythms inside the calculation window.

**Figure 6:**
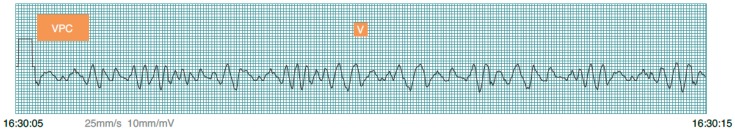
The misclassified VF rhythm to a VPC beat.

The next limitation is the algorithm sequence. Due to our study placing VF detection as the last evaluation, it may appear that VF wave and signal that is close to its wave are similar to the QRS complex classified as VPC class, as shown in Figure 6. This disadvantage is highly likely to be one of the factors negatively affecting VF detection.

For SVEB (i.e., APC) detection, according to ANSI/AAMI EC57, the evaluation should cover all data records. This condition will make a requirement to evaluate not only testing and validation data, but also training data of the ANN, which is learnt by the model in the training.

The device also has several limitations. For this system, the microcontroller unit speed is up to 25 MHz and the Bluetooth module will only support the data transferring maximum 2 Mbps data rates. In this study, utilizing single lead evaluation, these MCU and BLE still work very well. However, these conditions will make our device highly likely to have a problem for the multi-lead ECG signal evaluation.

For the electrode, this study utilizes a wet-based electrode. This electrode may have some disadvantages for the long-term user. The dry-up [50] and sweating [51] may affect the quality of the signal. In further, the utilization of the dry electrode will generate solutions for the wet electrode limitations in biopotential-based evaluation [52,53,54,55]. The dry electrode will be one of the future works for our study.

## 5. Conclusions

This study has developed an integrated method from several algorithms for arrhythmia detection by applying the relatively less complicated algorithms, which has purposed of the real-time wearable device for the arrhythmia detection. The system performs based on the R-R interval and the raw ECG signal for detecting the ECG abnormalities. This study evaluated 169 records from four databases in PhysioNet. Our study results for SVEB (i.e., APC) and VEB (i.e., VPC) have improved as compared to a previous study by utilizing the evaluation of ANSI/AAMI EC57:2012. For AF detection, most of the evaluations provide a positive achievement except for the episode sensitivity. Meanwhile, for VF, the episode sensitivity provides the decision from the whole databases ranging from 83% to 100%, except for the MITDB episode positive predictivity, which is 75%. In conclusion, our integrated algorithm detection can achieve a good accuracy in comparison to other previous studies. However, more advanced algorithms, faster MCU & BLE devices, and dry electrodes will be utilized as future works for our study. This will be a big advantage in solving data transfer problem and allow dry electrode multi-lead ECG system for more advanced arrhythmia detection and better evaluation.

## Figures and Tables

**Table 1:** The BC1 device specification.

CMRR (Common-mode rejection ratio)	80 dB (dc to 60 Hz)
High signal gain	(G = 100) with dc blocking capabilities
Single-supply operation	2.0 V to 3.5 V
ADC (Analog-to-Digital Converter)	12-bit
Input Impedance	5 Giga Ohm

**Table 2:** The entire supraventricular ectopic beat (SVEB) (i.e., APC) and ventricular ectopic beat (VEB) (i.e., VPC) evaluation result. (* = exclude records 2202, 8205; ** = exclude records 102, 104, 107, 217; N/A: not available. Se = Sensitivity, +P = Positive predictivity and FPR = False positive rate).

Database	Statistics	SVEB			VEB		
		Se	+P	FPR	Se	+P	FPR
AHADB *	Gross	N/A	N/A	N/A	89.75	96.08	0.371
	Average	N/A	N/A	N/A	86.52	84.67	0.458
MITDB **	Gross	79.87	67.14	1.323	93.10	95.65	0.321
	Average	71.35	36.9	2.098	87.27	73.26	0.336
NSTDB	Gross	N/A	N/A	N/A	83.22	45.79	10.180
	Average	N/A	N/A	N/A	58.17	50.86	9.032

**Table 3:** The entire atrial fibrillation (AF) and VF evaluation result. (* = exclude records 2202, 8205; ** = exclude records 102, 104, 107, 217; N/A: not available. ESe = Episode sensitivity, E + P = Episode positive predictivity, DSe = Duration sensitivity, D + P = Duration positive predictivity).

Database	Statistics	AF				VF			
		ESe	E + P	DSe	D + P	ESe	E + P	DSe	D + P
AHADB *	Gross	N/A	N/A	N/A	N/A	90	95	28	97
	Average	N/A	N/A	N/A	N/A	94	69	33	70
MITDB **	Gross	62	100	92	92	100	75	69	88
	Average	70	100	85	86	100	33	69	33
CUDB	Gross	N/A	N/A	N/A	N/A	83	90	32	94
	Average	N/A	N/A	N/A	N/A	84	83	40	84

**Table 4:** The SVEB (i.e., APC) and VEB (i.e., VPC) result comparison.

	Sensitivity (%)		Positive Predictivity (%)		False Positive Rate (%)	
	This Study	De Chazal et al. [44]	This Study	De Chazal et al. [44]	This Study	De Chazal et al. [44]
SVEB	79.87	75.9	67.14	38.5	1.323	4.7
VEB	93.1	77.7	95.65	81.9	0.321	1.2

**Table 5:** The AF and VF result comparison. (N/A = not available).

Arrhythmia	Studies	Database	Number of Data	Evaluation					
				Gross Statistics				Se	+P
				ESe	E + P	DSe	D + P		
Atrial Fibrillation	Proposed study	MITDB	44	62	100	92	92	N/A	N/A
	Young et al. [45]		12	N/A	N/A	N/A	N/A	97.7	86.77
Ventricular Fibrillation	Proposed study	AHADB	78	90	95	28	97	N/A	N/A
	Park et al. [46]		11	N/A	N/A	98.1	99.1	N/A	N/A
	Proposed study	MITDB	44	100	75	69	88	N/A	N/A
	Park et al. [46]		1	N/A	N/A	88.5	86.3	N/A	N/A
	Proposed study	CUDB	35	83	90	32	94	N/A	N/A
	Moraes et al. [47]		30	N/A	N/A	N/A	N/A	70.32	64.66

**Table 6:** The proposed integrated algorithm evaluation time in PC and smartphone for the MIT-BIH Arrhythmia database.

Record	Smartphone (s)	PC (s)	Record	Smartphone (s)	PC (s)
100	40.975	3.638	203	41.699	3.891
101	40.964	3.508	205	43.084	4.167
103	41.256	3.653	207	42.842	3.625
105	41.337	4.747	208	43.104	4.454
106	41.028	4.404	209	43.124	4.075
108	40.916	3.194	210	41.366	3.780
109	41.146	3.400	212	42.986	3.794
111	41.131	3.077	213	43.163	4.284
112	41.239	2.716	214	42.557	3.266
113	40.822	2.623	215	43.226	3.502
114	41.064	2.716	219	41.539	2.901
115	40.980	2.361	220	42.468	2.883
116	41.361	2.909	221	41.376	2.924
117	40.698	2.404	222	42.292	3.393
118	41.143	4.853	223	42.831	3.643
119	41.020	2.702	228	42.581	4.730
121	41.003	2.942	230	42.579	3.656
122	41.278	3.073	231	42.252	3.605
123	40.672	2.799	232	41.961	3.719
124	41.001	3.200	233	44.042	5.681
200	40.861	4.761	234	43.96	5.239
201	41.831	3.406	**Sum**	**1840.791**	**158.013**
202	42.033	3.718	**Mean**	**41.836**	**3.591**
			**STD**	**0.942**	**0.771**

**Table 7:** The study evaluation time comparison in personal computer (PC) and smartphone for the MIT-BIH Arrhythmia database. (N/A = not available).

Study	Arrhythmia	Device Evaluation Time (S)	
		Smartphone	PC
Proposed study	Normal, APC, VPC, AF, VF	1840.791	158.013
Chakroborty et al. [48]	Normal, APC, VPC, LBBB, RBBB	N/A	6875.3
